# ABIDE Delphi study: topics to discuss in diagnostic consultations in memory clinics

**DOI:** 10.1186/s13195-019-0531-y

**Published:** 2019-08-31

**Authors:** Agnetha D. Fruijtier, Leonie N. C. Visser, Ingrid S. van Maurik, Marissa D. Zwan, Femke H. Bouwman, Wiesje M. van der Flier, Ellen M. A. Smets

**Affiliations:** 1 Department of Neurology, Alzheimer Center Amsterdam, Amsterdam Neuroscience, Vrije Universiteit Amsterdam, Amsterdam UMC, Boelelaan 1118, 1081 HZ Amsterdam, The Netherlands; 2 Department of Medical Psychology, Amsterdam Public Health Research Institute, University of Amsterdam, Amsterdam UMC, Amsterdam, The Netherlands; 3 Department of Epidemiology and Biostatistics, Vrije Universiteit Amsterdam, Amsterdam UMC, Amsterdam, The Netherlands

**Keywords:** Informational needs, Information provision, Informative topics, Delphi consensus procedure, Memory clinics, Diagnostic process, Dementia, Mild cognitive impairment

## Abstract

**Background:**

Information given to patients and caregivers during the clinician-patient encounter varies considerably between memory clinic professionals. Patients and caregivers express a clear desire for more information. It is unclear what information patients and caregivers value most during the diagnostic process and whether this is concordant with professionals’ opinion. We aimed to identify a topic list on which health care professionals, patients, and caregivers agree that these should be discussed during diagnostic consultations in memory clinics. Further, we aimed to establish the optimal moment for each topic to be discussed during the diagnostic process.

**Methods:**

We performed a three-round Delphi consensus study. Professionals (*N* = 80), patients (*N* = 66), and caregivers (*N* = 76) rated the importance of 44 informative topics through an online questionnaire. Consensus was defined as a topic rating of 6 or 7 on a 7-point Likert scale by ≥ 75% of each panel. In round 2 and 3, a survey was added to identify the optimal moment during the diagnostic process to discuss each topic.

**Results:**

By round 3, consensus was achieved on 17 topics divided into four categories, information about (1) diagnostic testing, (2) test results, (3) diagnosis, and (4) practical implications. Eight additional topics showed significant differences between panels. Most notable panel differences regard the risk for developing dementia and the distinction between Alzheimer’s disease and dementia, which patients and caregivers evaluated as more important compared to professionals. The optimal moment to discuss topics during the diagnostic process was identified for the 17 core topics, and the eight topics with significant differences.

**Conclusions:**

We present a core list of informative topics, which professionals, patients, and caregivers agree they should be discussed during the diagnostic process in a memory clinic. The topic list can support professionals and empower patients and caregivers during diagnostic physician-patient consultations.

**Electronic supplementary material:**

The online version of this article (10.1186/s13195-019-0531-y) contains supplementary material, which is available to authorized users.

## Introduction

The diagnostic work-up of Alzheimer’s disease (AD) and other types of dementia is complex [1, 2]. Dementia develops gradually, there is considerable symptom variability between patients, and the ability to predict individual symptom progression is limited. Nowadays, many patients visiting a memory clinic are diagnosed with the syndrome diagnosis of mild cognitive impairment (MCI), a pre-dementia stage characterized by objective cognitive impairment not (yet) fulfilling criteria for dementia [3, 4]. New diagnostic tests, such as MRI, cerebrospinal fluid markers, and amyloid imaging using positron emission tomography can contribute to the diagnosis of neurodegenerative diseases underlying MCI or dementia, such as AD or other types of dementia [1, 2, 5]. In addition, these tests can help to better predict the progression of MCI to dementia [5, 6]. With the growing arsenal of diagnostic tests and a dawning insight that AD is a slowly progressive disease that starts long before onset of dementia, effective physician-patient communication about the diagnostic work-up for AD gains an importance [7–10]. Patients may vary in their views and expectations of the diagnostic process and also in the extent to which they want to be informed about the various options. Moreover, the increasing number of options does not always translate into increased certainty about the cause of the patients’ complaints. In a former study, we found that information provision during the physician-patient encounter varied considerably between memory clinic professionals [11–14]. Though most health care professionals reported informing their patients of all test results, patients and caregivers expressed a clear desire for more information about the results and the diagnosis [12].

Former studies have addressed which information patients and caregivers value most, but none assessed informational needs of patients and caregivers during the diagnostic process [15–20]. Moreover, none have compared this to the professionals’ opinion on which information needs to be provided. In addition, the informational needs of the caregiver are important in dementia and should be considered.

We designed the ABIDE Delphi study to gain insight in how health care professionals, patients, and caregivers value information about diagnostic tests, test results, diagnosis, and the implications of a diagnosis. Our objective was to identify a core set of informative topics on which health care professionals, patients, and caregivers agree that these should be discussed in physician-patient encounters during the diagnostic process, and identify differences between groups concerning which informative topics they value most.

## Methods

### Design

This study was part of the ABIDE project, an ongoing multicenter project investigating the clinical use of biomarkers in diagnostic testing in MCI and Alzheimer’s disease [21]. We performed a modified, three-round Delphi consensus study where participants rated through an online questionnaire the importance of discussing various informative topics in diagnostic consultations. The purpose of a Delphi study is to achieve group consensus by means of a structured, iterative communication process. By providing feedback about the answers in each new iteration, the group is informed of group development without direct group interaction, thus guaranteeing anonymity [22–24]. The study was reviewed by the board of the Medical Ethics Committee of the VU University Medical Center, Amsterdam UMC. All participants (professionals, patients, and caregivers) gave their informed consent digitally before participation, through an online registration form.

### Questionnaire development and Delphi process

We identified informative topics relevant for discussion in diagnostic memory clinic consultations from (1) (inter) national guidelines [1, 2, 5], (2) memory clinics’ information pamphlets on diagnostic tests, (3) patient letters containing information about the diagnostic process, (4) audio recordings of regular consultations, to (5) recorded focus groups of professionals, patients, and caregivers [11, 12]. We discussed a draft of the questionnaire with three physicians, two caregivers, and a patient. Subsequently, we tested the adjusted questionnaire in an online pilot with a new test panel of two physicians, three patients, and three caregivers. The participants in the pilot study did not participate in the final Delphi study. Their feedback was incorporated, resulting in the first version of the questionnaire, comprising 39 informative topics, divided into four categories; information on (1) diagnostic testing, (2) test results and (3) diagnosis, and (4) practical implications. Participants in round 1 received this first version of the questionnaire and were asked to rate the importance of each topic on a Likert scale ranging from 1: not important at all to 7: extremely important. In all rounds, participants had the opportunity to provide additional feedback in a free text field. As a result, five new topics were added to the questionnaire in round 2, resulting in a questionnaire of in total 44 topics (for the questionnaire topics, see Additional file 1). In rounds 2 and 3, participants were also asked to report their preferred moment(s) for discussion for each informative topic; (1) before testing in a consult with the physician, (2) after testing, when receiving results in a consult with the physician, and/or (3) some time after receiving the diagnosis in a consult with someone other than the physician (i.e., a nurse, the patients’ general practitioner, or a case manager).

**Fig. 1:**
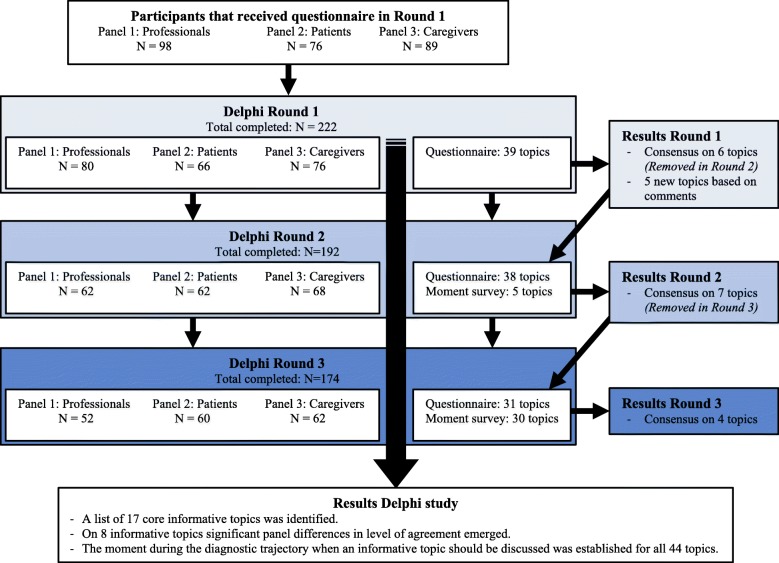
Flowchart of the ABIDE Delphi process

After round 1, each following round participants received feedback of the answers of the total group during the previous round (for the Delphi study flow chart, see Fig. 1). Participants were only invited for the next round if they had completed the questionnaire during the previous round. Topics on which consensus was reached were removed from the next iteration.

Consensus was reached when a topic was rated in the top or bottom two scores of the 7-point Likert scale by the majority of participants in each panel (≥ 75%). A core topic was defined as a topic on which consensus was achieved in the top two scores of the Likert scale (6–7), thus identifying the topic as “very” or “extremely” important. Primary moment was determined by the majority of the total group (≥ 50%) selecting that moment for a topic.

### Panels

The three panels for the Delphi study consisted of (1) memory clinic professionals (professionals), (2) patients who had visited a memory clinic for their cognitive complaints, and (3) caregivers of memory clinic patients (not necessarily related to the participating patients). Professionals were recruited from the ABIDE project group, through an advertisement in the newsletter of the Dutch Memory Clinic Network and by direct mail, and included neurologists, geriatricians, internist-geriatricians, nurses, (neuro) psychologists, and psychiatrists.

Patients were eligible if they had received a diagnosis of early-stage dementia, MCI, or subjective cognitive decline (SCD) no longer than 1.5 years ago. Caregivers could participate if they had been present during a visit to a memory clinic (accompanying a patient) no longer than 5 years ago. Patients and caregivers were recruited through the website of the Dutch Alzheimer association “Alzheimer Nederland;” an online platform for patients and caregivers “dementie.nl” and social media; the Amsterdam Dementia Cohort [25]; and advertisements or direct mail in five memory clinics. Caregivers were also recruited through the online participant recruitment registry Hersenonderzoek.nl (www.hersenonderzoek.nl). All participants were required to be able to read and write Dutch.

There are no guidelines on the sample size for Delphi studies, though numbers between 10 and 50 are often suggested [22–24]. To ensure sufficient variation within each panel, we aimed to include 75 participants in each of the three panels.

### Statistics

Data were analyzed using version 22.0 of SPSS for Windows. Frequencies, median, and interquartile range were calculated for level of agreement within each panel. Group differences were evaluated by means of Kruskal-Wallis test.

### Delphi meetings

Following the online questionnaire, we conducted two in-person meetings with a sample of participants with the aim to explore (1) professionals’, patients’, and caregivers’ opinions regarding differences between panels on informative topics and (2) the timing of information provision. The first meeting was attended by three physicians, four patients, and five caregivers and the second meeting by one physician, two patients, and two caregivers. Each meeting started with a brief presentation of the results of the online Delphi questionnaire followed by a group discussion. Both meetings were audio recorded, and the recordings were independently summarized and content-coded by two coders (A.F. and I.v.M.).

## Results

### Panel characteristics

Initially, 98 professionals, 76 patients, and 89 caregivers registered for participation. Round 1 was completed by 80 professionals, 66 patients, and 76 caregivers from 61 different memory clinics, from both academic and local hospitals (panel demographic data presented in Table 1). Finally, 52 professionals (52 of 80 who completed round 1; 65%), 60 patients (60/66; 91%), and 63 caregivers (63/76; 83%) completed all three rounds. Participation level within each round is shown in Fig. 1.

**Table 1:** Participant characteristics

Characteristic	Panel 1: Professionals	Panel 2: Patients	Panel 3: Caregivers
*N*	80^†^	66^†^	76^†^
Age	46 (± 10)	65 (± 8)	60 (± 9)
Female	55 (69%)	25 (38%)	62 (82%)
Experience (years)	10 (± 7)	n.a.	n.a.
Profession			
Neurologist	23 (29%)	n.a.	n.a.
(Internist-) geriatrician	24 (30%)	n.a.	n.a.
Nurse (specialist)	17 (21%)	n.a.	n.a.
Psychologist/psychiatrist	14 (17%)	n.a.	n.a.
Other	2 (3%)	n.a.	n.a.
Education level^‡^			
Low	0 (0%)	14 (21%)	15 (20%)
Intermediate	7 (9%)	13 (20%)	25 (33%)
High	73 (91%)	39 (59%)	36 (47%)
Time since diagnosis*	n.a.	0.9 (± 1)	1.4 (± 1)
Diagnosis*			
Cognitively normal/SCD	n.a.	13 (20%)	0 (0%)
MCI	n.a.	13 (20%)	4 (5%)
Dementia	n.a.	30 (45%)	63 (83%)
Other	n.a.	10 (15%)	9 (12%)
Hospital			
Academic	26 (33%)	40 (61%)	18 (24%)
Local	54 (67%)	26 (39%)	58 (76%)

NOTE. *For the caregiver group, numbers represent (time since) diagnosis of their loved ones

†Number of participants who completed round 1

‡Education level index according to the Dutch National standard education index (SOI)

### Core set of informative topics

**Fig. 2:**
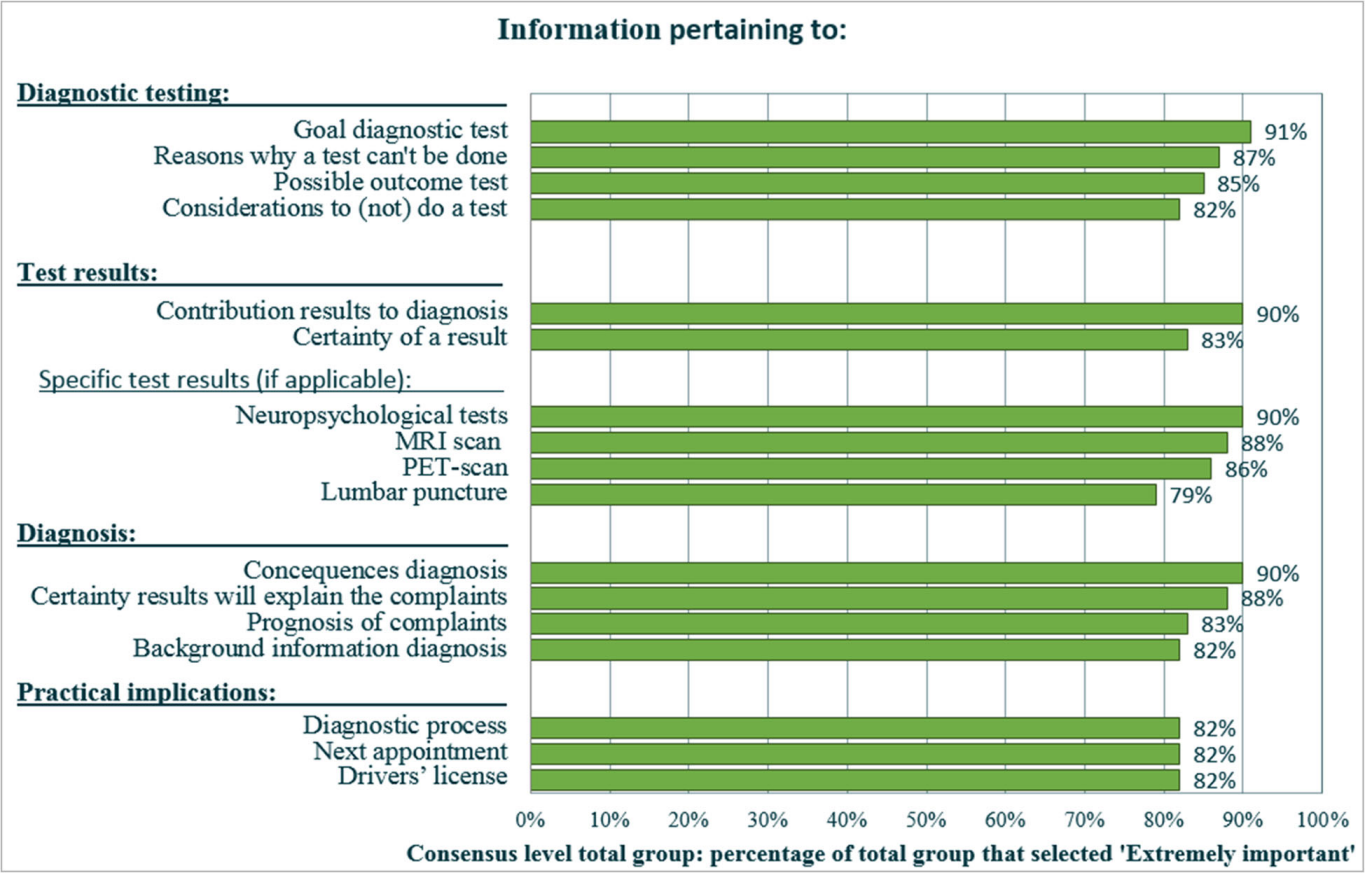
Core set of informative topics on which consensus was reached in all three panels. NOTE. Consensus was reached if a topic was rated 6 or 7 on the Likert scale (“extremely important”) by 75% within each group (professionals, patients, and caregivers)

After three rounds, all 44 informative topics were valued at least as moderately important by all three panels (5 or higher on the Likert scale). No topics were uniformly valued as unimportant (3 or lower on the Likert scale). Hence, none of the 44 informative topics were excluded in the following rounds. Figure 2 shows the core set of 17 informative topics that were considered important by all three panels (6 or 7 on the Likert scale). The 17 core informative topics represented the four questionnaire categories (diagnostic tests, test results, diagnosis, and practical implications). We observed remarkably high consensus (≥ 90%) for the following four topics from the core set; information about (1) the goal of a diagnostic test (i.e., what we can find out using this test), (2) the results of neuropsychological tests, (3) the contribution of individual test results to a diagnosis, and (4) consequences of the diagnosis.

### Commentary in free text fields

A noteworthy observation in the commentary was that 20 of the 80 professionals (25%) noted that situational context (e.g., a particular diagnosis, age, or specific health care demand), as well as patients’ or caregivers’ capacity to understand, should be considered when providing information.

### Panel differences

Professionals generally rated the importance of topics somewhat lower than both patients and caregivers. We observed that in general, professionals disagreed more with other professionals on the relative importance of informative topics than patients and caregivers.

**Fig. 3:**
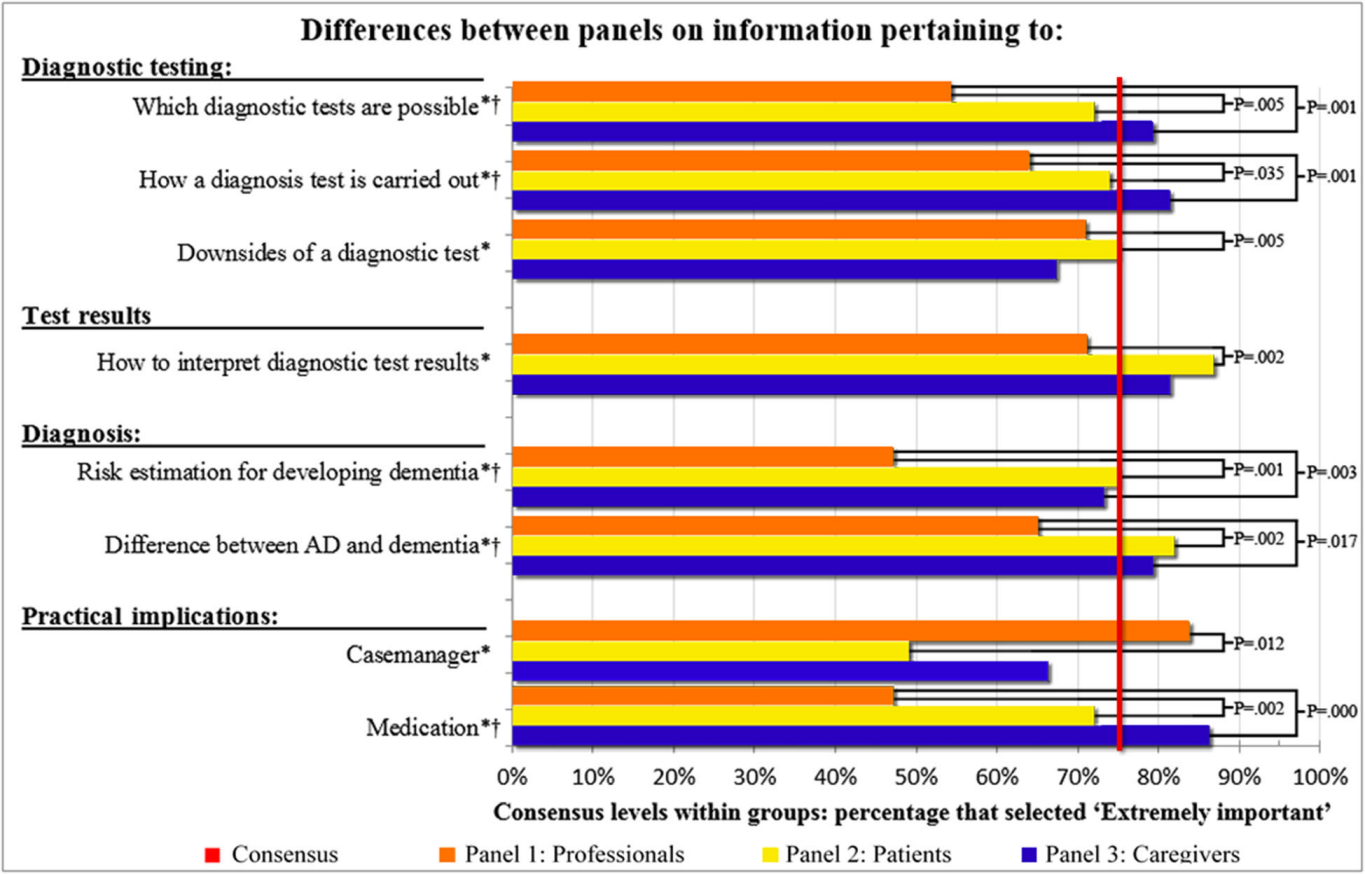
Eight topics with significant differences between the panels’ agreement levels. NOTE. *Significant difference between professionals and patients (*P* value < 0.05). †Significant difference between professionals and caregivers (*P* value < 0.05)

Eight informative topics were rated as extremely important by one or two of the panels, but differed significantly from another panel (*P* values ≤ .05, Fig. 3). Patients and caregivers deemed “difference between Alzheimer’s disease and dementia,” “risk estimation for developing dementia,” “which diagnostic tests are possible” and “information about medication,” “how a test is carried out,” “downsides of a test,” and “how to interpret a test result” more important than professionals. Conversely, the topic “practical information about a case manager” received higher ratings from professionals as compared to the other two panels. In the Netherlands, a case manager is a nurse specialized in dementia care who coordinates care services and to serve as a primary contact for the family of a person with dementia.

### Delphi meetings

Regarding the eight topics for which opinions between panels in the online questionnaire diverged, the in-person meetings revealed that these differing opinions originated mostly from opinions on the extent to which, i.e., the “how” and “when” a topic should be discussed rather than “if” it should be discussed. A clear preference to receive information on these topics emerged amongst patients and caregivers. Participants argued that, although some information did not necessarily provide more certainty about their future, it would increase their knowledge about the cause of their complaints. Such knowledge would provide relief of their uncertainty and a framework from which to make decisions regarding their care and future. Further, participating patients and caregivers underscored the importance of information, even if that information might be unsettling. While participating professionals supported these arguments, they also underscored the difficulty of discussing these issues.

### Moment of providing information

**Fig. 4:**
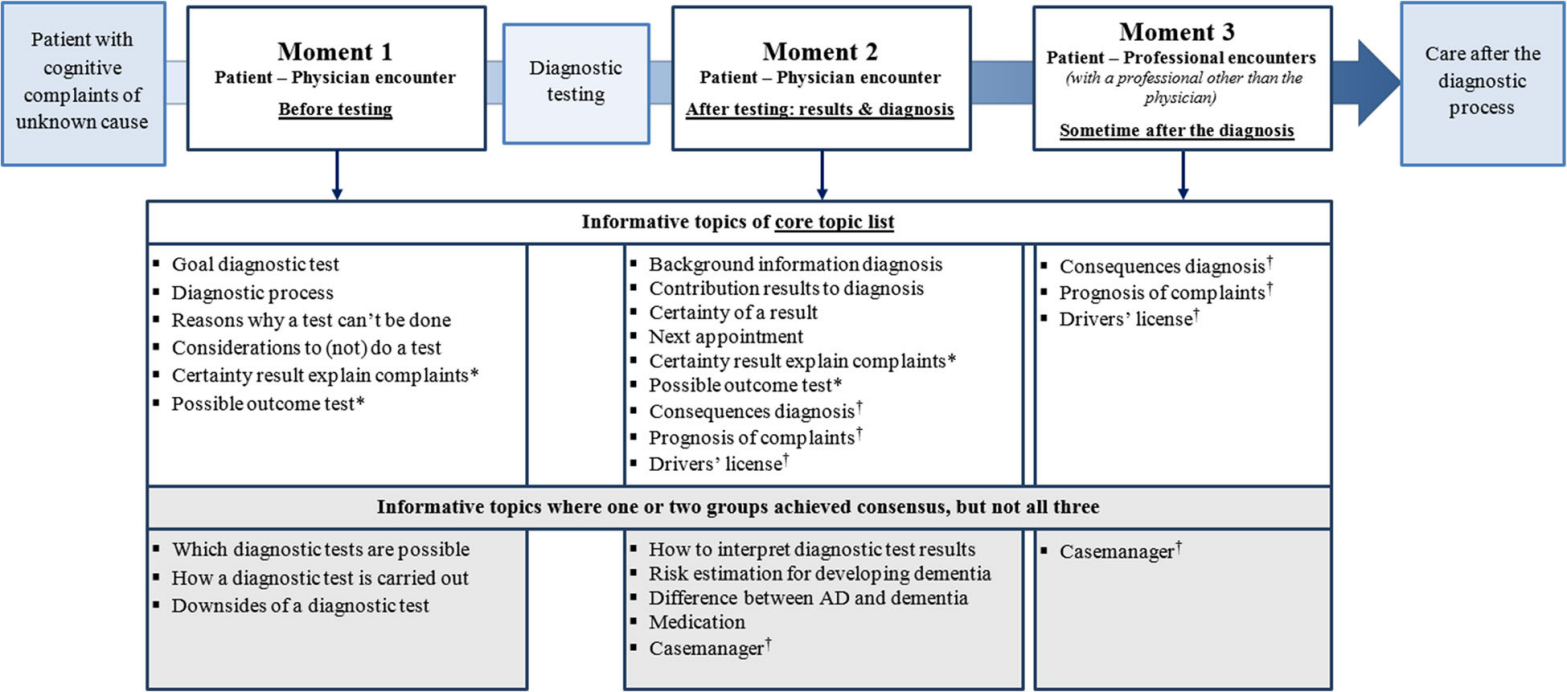
When to discuss topics during the diagnostic process. NOTE. *The majority selected both moment 1 and moment 2 for this topic. †The majority selected both moment 2 and moment 3 for this topic

Figure 4 shows the results of questions addressing the preferred moment of discussing topics in the diagnostic process. For most core topics, the majority of participants indicated either before testing (moment 1) or after testing when disclosing results (moment 2) as the optimal moment. For two topics, both of these encounters were considered as relevant (the topics “certainty that test results will explain the complaints” and “the possible outcomes of a test”). Furthermore, for three topics (“consequences of the diagnosis,” “prognosis of the symptoms,” and “information about the drivers’ license”), participants considered it important to repeat this information sometime after receiving the test results, in a consultation with another health care professional than the physician. In addition, for topics with differences between groups, the majority of participants considered either before testing (moment 1) or when disclosing results (moment 2) the optimal moment. Here, only for the topic “case manager” did participants consider the moment after testing (moment 2) and some time after diagnosis (moment 3) relevant.

## Discussion

The ABIDE Delphi study identified a list of 17 informative topics on which professionals, patients, and caregivers agree that these should be discussed in the consulting room during the diagnostic process in a memory clinic. This core topic list, supplemented with the moment at which information should be discussed, can support professionals and empower patients and caregivers to get the most value from diagnostic physician-patient consultations.

During the pre-test consultation, the physician could use the list to enquire about the patients’ informational needs regarding, e.g., the goal of a test, possible test results, or the certainty of those results. Additionally, the topic list could serve as a means of education if it were incorporated into an informational brochure provided to patients and caregivers prior to their visit. The list would allow patients or caregivers to become aware of potentially relevant knowledge gaps prior to a visit, look up information to discuss with their physician, or simply enhance their understanding of different facets of the diagnostic process. During the post-test consultation, a patient may become aware that, aside from hearing the diagnosis, they would like information on the certainty or the consequences of a diagnosis. Patients or caregivers could then use the topic list prior to their visit to formulate questions to ask their physician.

We identified eight topics which are deemed extremely important by at least one, but not all three groups. Hence, it is recommended to address these topics in consultations when applicable and/or ask whether the patient or caregiver wishes to receive this information. When examining the general differences between the panels, it is noticeable that professionals generally attributed less importance to informative topics than both the patients and the caregivers. Their insight into daily procedures in their memory clinic, including the limited consultation time, is a likely explanation for this difference. A notable exception is information provision about the availability and role of the case manager, which professionals rated as significantly more important than either patients or caregivers. The Delphi meetings suggest that this difference in perspective is likely the result of the benefits and purpose of a case manager still being largely unknown to patients and their caregivers. Further, our results show that professionals, patients, and caregivers highly value providing or receiving individual diagnostic test results, yet we did observe a difference in rating of importance between the various diagnostic tests. We believe this may be attributed in part to the evaluation of the professionals, who have clinical insight into the diagnostic tests and their value for a diagnosis. Patients and caregivers may have evaluated discussion of individual diagnostic test result as extremely important, assuming the said tests would be done, whereas professionals may have taken the rarer use of certain diagnostic tests into consideration in their evaluation of their importance. It is also possible that a neuropsychological examination and MRI scan are perhaps simply the most well-known diagnostic tests to patients and caregivers, as these tests are used most often in the diagnostic process.

A noteworthy theme that emerged during the meetings was relief of uncertainty. Patients and caregivers indicated that any answer providing insight into what was wrong gave them some relief of uncertainty. This was considered true even for information with a high degree of uncertainty itself, such as a risk estimation. Here, it seems that the benefits of having even a partial answer to questions about the cause of their complaints or what to expect in the future, outweighs the downsides of knowing that information. In the meetings, this appeared to be of particular importance for information regarding etiology and risk estimations for developing dementia. This result is concordant with earlier findings that report information on what to expect is amongst the most important topics for caregivers of persons with dementia [15].

Existing studies into informational needs amongst dementia patients focus on the time after diagnosis, a specific subset of the patient population or later stages of dementia such as hospice care [15–19]. Although their findings all concern information after having received the diagnosis, they too found a consistent need for information focused on future expectations or practical implication amongst patients and caregivers. Our study did not make this distinction and does not only provide insight into which informational topics are considered most important by patients and caregivers, but also to what degree these views are concordant with professionals’ views on information provision. We included all three groups in our sample and therefore our results are supported by professionals, patients, and caregivers. Herein lies the main strength of our study.

However, the design of our study also has its limitations. For one, considering the abstract nature of the questions and the concept of the Delphi method, we did not include patients with more advanced stages of dementia. As a result, our results do not include the views of these patients. In addition, inclusion was limited to Dutch memory clinics. Nonetheless, the topics in the core list (Fig. 2) concern diverse aspects of the diagnostic process, which have worldwide relevance, beyond the Dutch setting. Of note, not only is there practice variation in the diagnostic process internationally, there is also practice variation amongst Dutch memory clinics [11–14]. To ensure this variation was mirrored in our data, we included participants (patients, caregivers, professionals) from 61 different hospitals nationwide, including academic and local hospitals. We believe that this broad representation of variable clinical practice adds to the generalizability of the results beyond the Dutch borders. Nonetheless, some of the topics included in our study might be more relevant in the Dutch setting (e.g., information about the case manager), while topics not included in our study could be more relevant in other settings (e.g., financial implications). Therefore, future research is warranted to validate the ABIDE core topic list in other countries and in other populations, such as the general older population at risk of dementia, who might in the future benefit from preventive strategies. Further, a quarter of our participating professionals noted the importance of patient context in information provision. While our patient and caregiver groups were quite large for the purpose of the study, they were unsuitable to assess differences based on patient or caregiver characteristics within either group (e.g., diagnosis or age). In addition, while our live meetings offered valuable insights into group differences, these insights focused on the importance of specific informative topics, rather than on the nature of information patients and/ or caregivers desired on these topics (which information about, for example, medication was desired). Understanding differences could benefit tailoring information to individual informational needs, as well as aid the development of educational material directed at patients or caregivers specifically. As such, additional research is needed to better understand the differences between, and amongst, patients and caregivers. Finally, we observed stronger opposing views on informational topics within the group professionals. Again, our study was unsuited to provide a more in-depth look at these different points of view within the professionals’ group. In light of the existing practice variation, more in-depth research into these differences amongst professionals would be an interesting potential avenue for future research. Despite these limitations, the results of the Delphi study provide an evidence-based advice for information provision in memory clinics.

## Conclusion

We present a core list of informative topics, on which professionals, patients, and caregivers agree they should be discussed during the diagnostic process in a memory clinic. If implemented, the topic list may be useful in supporting professionals in their communication with patients and caregivers. In addition, it will empower patients and caregivers in their preparation for and communication during diagnostic consultations.

## Additional file

Additional file 1: The 44 informative topics in the Delphi questionnaire. The 44 informative topics in the Delphi questionnaire. Complete list of all 44 informative topics included in the Delphi questionnaire. (PDF 209 kb)

## Data Availability

The datasets used during the current study are available from the corresponding author on reasonable request.

## Abbreviations

AD: Alzheimer’s disease
MCI: Mild cognitive impairment
SCD: Subjective cognitive decline

## References

[1] Scheltens P, Blennow K, Breteler MMB, de Strooper B, Frisoni GB, Salloway S (2016). Alzheimer’s disease. Lancet.

[2] Dubois B, Hampel H, Feldman HH, Scheltens P, Aisen P, Andrieu S (2016). Preclinical Alzheimer’s disease: definition, natural history, and diagnostic criteria. Alzheimers Dement.

[3] Albert MS, DeKosky ST, Dickson D, Dubois B, Feldman HH, Fox NC (2011). The diagnosis of mild cognitive impairment due to Alzheimer’s disease: recommendations from the National Institute on Aging-Alzheimer’s Association workgroups on diagnostic guidelines for Alzheimer’s disease. Alzheimers Dement.

[4] Petersen RC, Lopez OL, Armstrong MJ, Getchius TSD, Ganguli M, Gloss D, et al. Practice guideline update: Mild cognitive impairment. American Academy of Neurology. 2017. https://www.aan.com/Guidelines/home/GuidelineDetail/881. Accessed 3 Mar 2018.

[5] Jansen WJ, Ossenkoppele R, Knol DL, Tijms BM, Scheltens P, Verhey FRJ (2015). Prevalence of cerebral amyloid pathology in persons without dementia. JAMA..

[6] van Maurik IS, Zwan MD, Tijms BM, Bouwman FH, Teunissen CE, Scheltens P (2017). Interpreting biomarker results in individual patients with mild cognitive impairment in the Alzheimer’s Biomarkers in Daily Practice (ABIDE) project. JAMA Neurol.

[7] Dubois B, Padovani A, Scheltens P, Rossi A, Dell’Agnello G, Saykin A (2015). Timely diagnosis for Alzheimer’s disease: a literature review on benefits and challenges. J Alzheimers Dis.

[8] Grill JD, Apostolova LG, Bullain S, Burns JM, Cox CG, Dick M, et al. Communicating mild cognitive impairment diagnoses with and without amyloid imaging. Alzheimers Res Ther. 2017;9(1):35. 10.1186/s13195-017-0261-y. 10.1186/s13195-017-0261-y PMC5418690 28472970

[9] Visser PJ, Wolf H, Frisoni G, Gertz H (2012). Disclosure of Alzheimer's disease biomarker status in subjects with mild cognitive impairment. Biomarkers Med.

[10] van der Flier WM, Kunneman M, Bouwman FH, Petersen RC, Smets EMA (2017). Diagnostic dilemmas in Alzheimer’s disease: room for shared decision making. Alzheimers Dementia.

[11] Kunneman M, Pel-Littel R, Bouwman FH, Gillissen F, Schoonenboom NSM, Claus JJ (2017). Patients’ and caregivers’ views on conversations and shared decision making in diagnostic testing for Alzheimer’s disease: the ABIDE project. Alzheimers Dementia.

[12] Kunneman M, Smets EMA, Bouwman FH, Schoonenboom NSM, Zwan MD, Pel-Littel R (2017). Clinicians’ views on conversations and shared decision making in diagnostic testing for Alzheimer's disease: the ABIDE project. Alzheimers Dementia.

[13] Visser Leonie N.C., Kunneman Marleen, Murugesu Laxsini, van Maurik Ingrid, Zwan Marissa, Bouwman Femke H., Schuur Jacqueline, Wind Hilje A., Blaauw Marjolijn S.J., Kragt J.Jolijn, Roks Gerwin, Boelaarts Leo, Schipper Annemieke C., Schooneboom Niki, Scheltens Philip, van der Flier Wiesje M., Smets Ellen M.A. (2019). Clinician-patient communication during the diagnostic workup: The ABIDE project. Alzheimer's & Dementia: Diagnosis, Assessment & Disease Monitoring.

[14] Kunneman M, Bouwman FH, Smets EMA, van der Flier WM (2018). Diagnostiek van dementie: praktijkvariatie in Nederlandse geheugenpoliklinieken. Neuropraxis..

[15] Wackerbarth SB, Johnson MMS (2002). Essential information and support needs of family caregivers. Patient Educ Couns.

[16] Casarett D, Takesaka J, Karlawish J, Hirschman KB, Clark CM (2002). How should clinicians discuss hospice for patients with dementia? Anticipating Caregivers’ preconceptions and meeting their information needs. Alzheimer Dis Assoc Disord.

[17] Edelman P, Kuhn D, Fulton BR, Kyrouac GA (2006). Information and service needs of persons with Alzheimer’s disease and their family caregivers living in rural communities. Am J Alzheimers Dis Other Dement.

[18] Nichols LO, Martindale-Adams J, Greene WA, Burns R, Graney MJ, Lummus A (2008). Dementia caregivers’ most pressing concerns. Clin Gerontol.

[19] Washington KT, Meadows SE, Elliott SG, Koopman RJ (2011). Information needs of informal caregivers of older adults with chronic health conditions. Patient Educ Couns.

[20] Whitlatch CJ, Orsulic-Jeras S (2017). Meeting the informational, educational, and psychosocial support needs of persons living with dementia and their family caregivers. The Gerontologist.

[21] de Wilde A, van Maurik IS, Kunneman M, Bouwman F, Zwan M, Willemse EAJ (2017). Alzheimer’s biomarkers in daily practice (ABIDE) project: rationale and design. Assess Dis Monit.

[22] Hasson F, Keeney S, McKenna H (2000). Reasearch guidelines for the Delphi survey technique. J Adv Nurs.

[23] Murry JW, Hammons JO (1995). Delphi a versatile methodology for conducting qualitative research. Rev High Educ.

[24] Hsu C, Sandford BA. The Delphi technique, making sense of consensus. Pract Assess Res Eval. 2007;12(10). http://pareonline.net/pdf/v12n10.pdf. Accessed 23 May 2017.

[25] van der Flier WM, Scheltens P, Perry G, Avila J, Tabaton M, Zhu X (2018). Amsterdam dementia cohort: performing research to optimize care. J Alzheimers Dis.

