# Supplementary material for: ABIDE Delphi study: topics to discuss in diagnostic consultations in memory clinics
Source: Alzheimers Res Ther. 2019 Aug 31;11:77. doi: 10.1186/s13195-019-0531-y (PMC6717649; doi:10.1186/s13195-019-0531-y)
Supplement: Supplementary file 1 — The 44 informative topics in the Delphi questionnaire. The 44 informative topics in the Delphi questionnaire. Complete list of all 44 informative topics included in the Delphi questionnaire. (PDF 209 kb) [file 13195_2019_531_MOESM1_ESM.pdf]

Additional file 1
Complete list of all 44 informative topics included in the questionnaire.

Information on diagnostic testing

1. Which tests are possible
2. How a test is carried out
3. How long a test will take
4. Possible benefits of a test
5. Possible harms of a test
6. Reasons why a test can't be done
7. Goal diagnostic test
8. Possible outcome test
9. Doing additional diagnostic tests
10. Possibility of not testing
11. Physicians' considerations to (not) do a test

Information on the test results

12. Contribution results to diagnosis
13. Certainty of a result
    ('With the results of this test we know with certainty it is not Alzheimer's disease')
14. How to interpret the results
15. Results for each separate diagnostic test
16. Importance of what caregiver and patient tell about the complaints

Specific test results:

17. General physical examination
18. Bloodwork
19. Neuropsychological tests
20. MRI-scan
21. CT-scan
22. PET-scan
23. EEG
24. Lumbar Puncture

Information on the diagnosis

25. Certainty results will explain the complaints
    ('With these tests we want to find out what the cause for your complaints is, however it is possible we won't know more about the cause after doing this test')
26. Background information diagnosis
27. Prognosis of complaints
28. Consequences diagnosis
29. Risk estimation for developing dementia
30. Difference between Alzheimer's disease and dementia

Practical implications

31. Information about the diagnostic process
32. Planning of the diagnostic tests
33. Casemanager
34. Drivers' license
35. Advice work situation
36. Medication
37. Lifestyle advice
38. Genetic testing
39. Next appointment
40. Feedback to the primary physician
41. Information about other care services
42. Participation in scientific research
43. Feedback to other care services
44. Legal matters
